# Supplementary figures and images for: Interplay between Pitx2 and Pax7 temporally governs specification of extraocular muscle stem cells
Source: PLoS Genet. 2024 Jun 14;20(6):e1010935. doi: 10.1371/journal.pgen.1010935 (PMC11178213; doi:10.1371/journal.pgen.1010935)

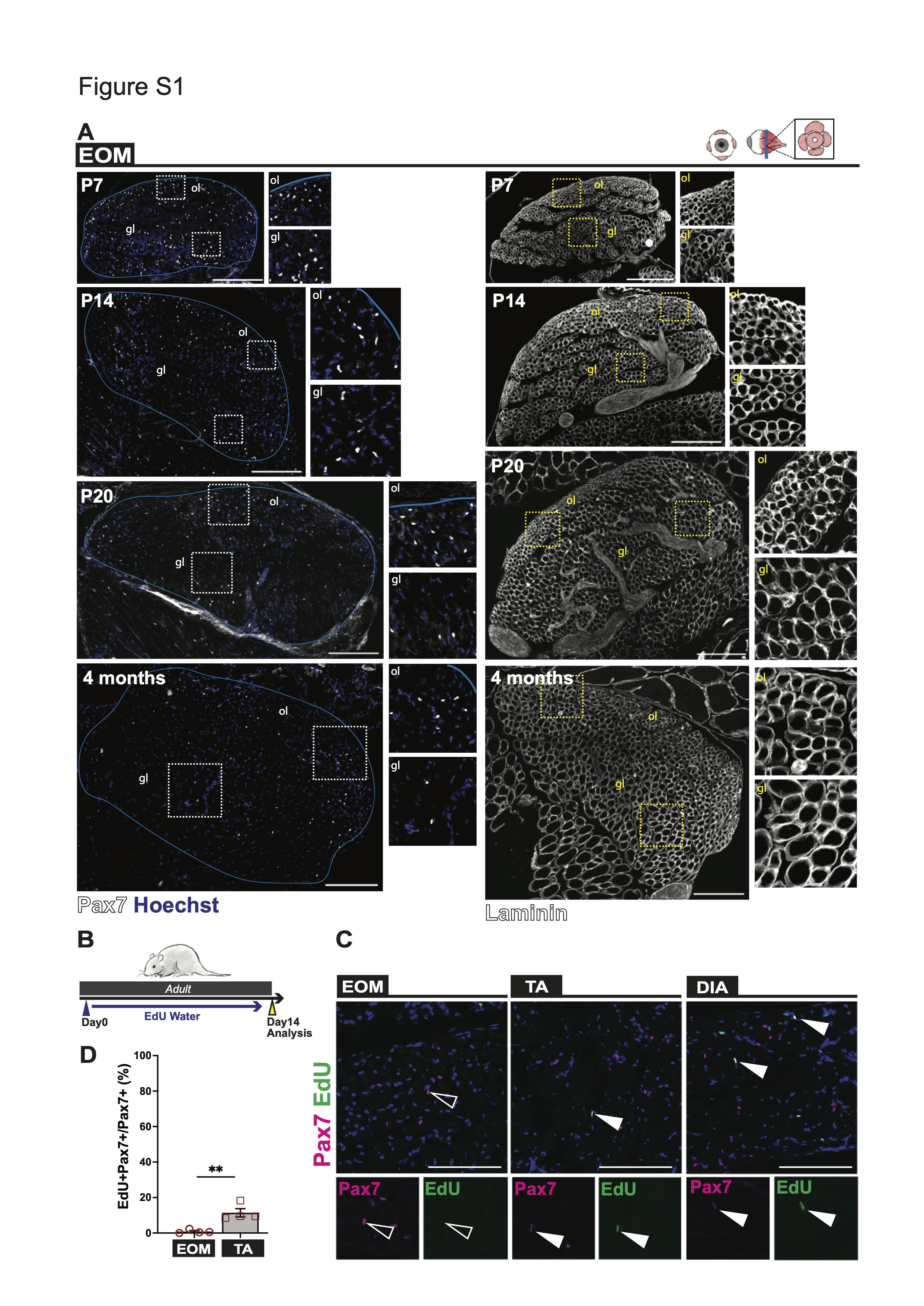

Supplement: S1 Fig — (A) Immunostaining of P7, P14, P20 and 4 months old EOM sections for (A) Pax7 (white) and Hoechst nuclei staining or (B) Laminin (white). Global layer (gl) and orbital layer (ol). Higher magnification views of the areas delimited with dots. (B) Scheme of the experiment. EdU was administered in drinking water to adult mice for 2 weeks. (C) Immunostaining for Pax7 and EdU detection at the EOM, TA, and DIA level as per the experiment in B. White arrowheads indicate Pax7+EdU+ cells. White open arrowheads indicate Pax7+EdU- cells. (D) Percentage of EdU+Pax7+ cells over total Pax7+cells on EOM and TA sections (n = 4 each). Scale bars: 200μm in (A), 100μm in (C). Error bars represent mean ± SEM. Two-tailed unpaired Student’s t-test. **P<0.01. EOM, extraocular muscle; TA, Tibialis anterior; DIA, diaphragm. All recti EOMs were assessed. (JPG) [file pgen.1010935.s001.jpg]

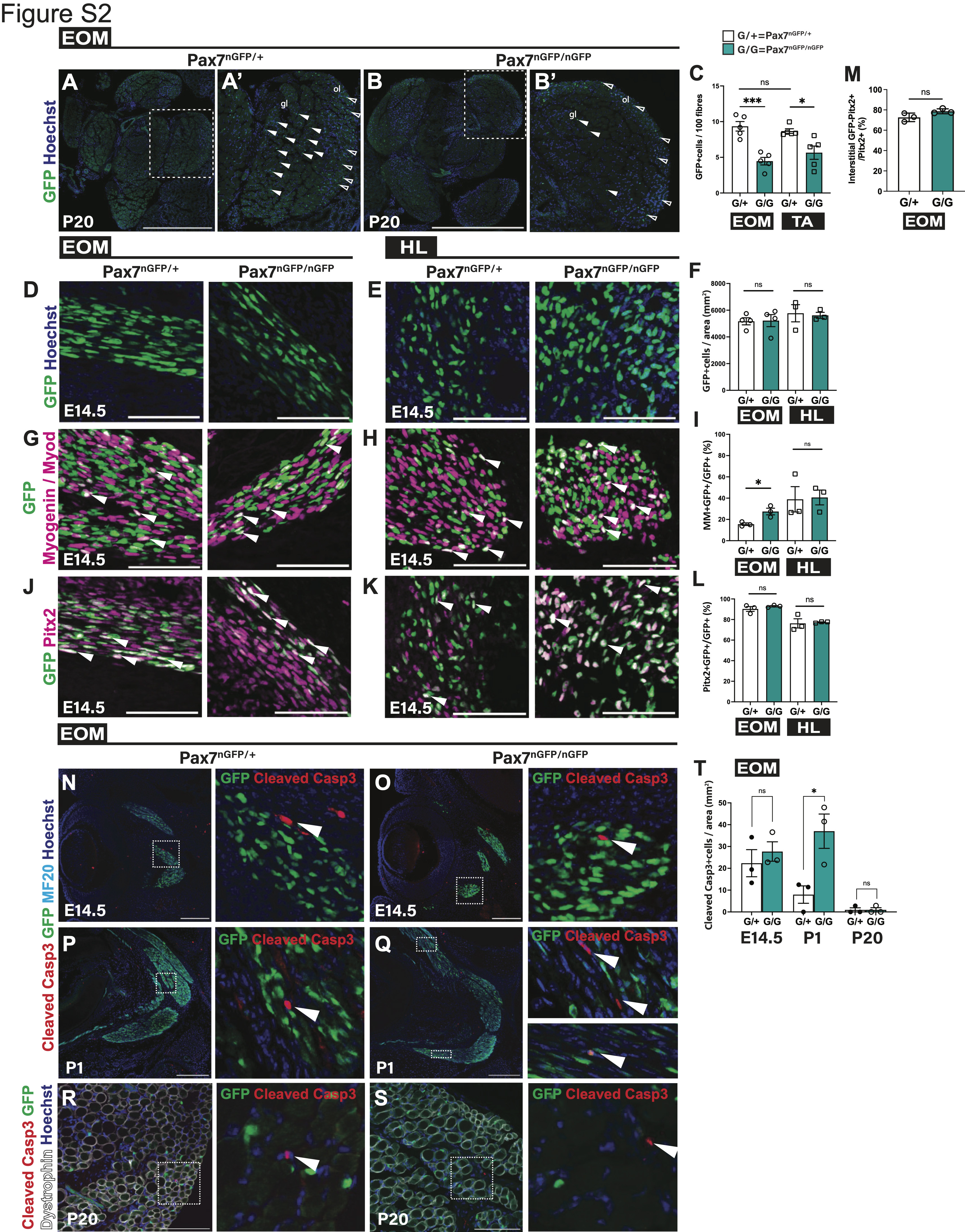

Supplement: S2 Fig — (A, B) Immunostaining for GFP (green) on EOM sections from Pax7nGFP/+ and Pax7nGFP/nGFP mice at P20. (A’, B’) Higher magnification views of the area delimited with dots. White arrowheads indicate GFP+ cells in global layer (gl); open arrowheads indicate GFP+ cells in orbital layer (ol). (C) Number of GFP+ cells per 100 fibres at P20 (n = 5 each). (D,E) Immunostaining for GFP (green) on EOM and HL sections from Pax7nGFP/+ and Pax7nGFP/nGFP mice at E14.5. (F) Number of GFP+ cells per area from immunostaining in (D,E) (EOM n = 4, TA n = 3). (G,H) Immunostaining for GFP (green) together with Myod and Myogenin (MM) (magenta) on EOM and HL sections from Pax7nGFP/+ and Pax7nGFP/nGFP mice at E14.5. White arrowheads indicate MM+GFP+ cells. (I) Percentage of MM+GFP+ cells over total GFP+ population from immunostaining in (G,H) (n = 3 each). (J, K) Immunostaining for GFP (green) and Pitx2 (magenta) on EOM and HL sections from Pax7nGFP/+ and Pax7nGFP/nGFP mice at E14.5. White arrowheads indicate Pitx2+GFP+ cells. (L) Percentage of Pitx2+GFP+ cells over total GFP+ population from immunostaining in (J,K) (n = 3 each). (M) Percentage of GFP-Pitx2+ cells over the total number of Pitx2+ interstitial cells on EOM sections at P20 (n = 3 each). Laminin was used for counting cells in the interstitium. (N-Q) Immunostaining for GFP (green), cleaved-caspase3 (Red) and MF20 (cyan) at the level of the EOMs from Pax7nGFP/+ and Pax7nGFP/nGFP mice at E14.5 (N,O) and P1 (P,Q). Higher magnification views of the area delimited with dots. White arrowheads indicate Cleaved-Caspase3+ cells. (R, S) Immunostaining for GFP (green), Cleaved-Caspase3 (Red) and dystrophin (white) on EOM sections from Pax7nGFP/+ and Pax7nGFP/nGFP mice at P20. Higher magnification views of the area delimited with dots. White arrowheads indicate Cleaved-Caspase3+ cells. (T) Number of Cleaved-Caspase3+ cells per area from immunostaining in (N-S) (n = 3 per stage). G/+: Pax7nGFP/+, G/G: Pax7nGFP/nGFP. Scale bars: 1000μm in (A, [file pgen.1010935.s002.jpg]

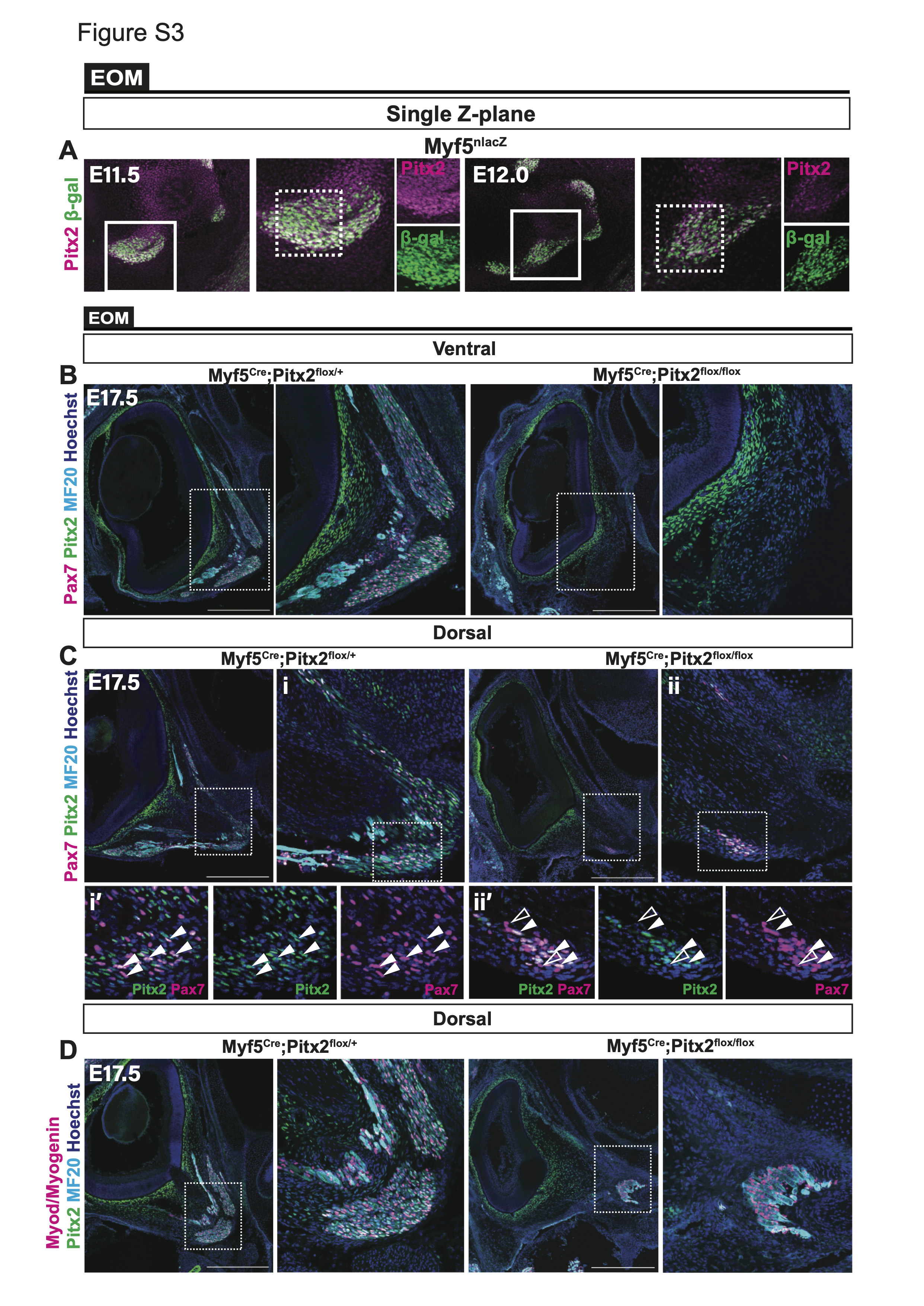

Supplement: S3 Fig — (A) Single Z-section of the respective WMIF segmented volumes of whole-mount immunostaining of Myf5nlacZ EOM anlage for Pitx2 (magenta) and β-gal (green) at E11.5, E12.0 from Fig 3B. Right panels, higher magnification views of the area delimited with white line or dots. (B,C) Immunostaining of EOM sections from Myf5Cre;Pitx2flox/+ (control) and Myf5Cre;Pitx2flox/flox (KO) at E17.5 for Pax7 (magenta), Pitx2 (green) and MF20 (cyan). White arrowheads indicate Pax7+Pitx2+ cells; open arrowheads indicate Pax7+Pitx2- cells. (D) Immunostaining of EOM sections from Myf5Cre;Pitx2flox/+ (control) and Myf5Cre;Pitx2flox/flox (KO) at E17.5 for Myod and Myogenin (magenta), Pitx2 (green) and MF20 (cyan). Higher magnification views of the area delimited with dots. Samples were evaluated in ventral (B) and dorsal (C,D) anatomical locations. Scale bars: 500μm (B-D). EOM, extraocular muscle. All recti EOMs were assessed. (JPG) [file pgen.1010935.s003.jpg]

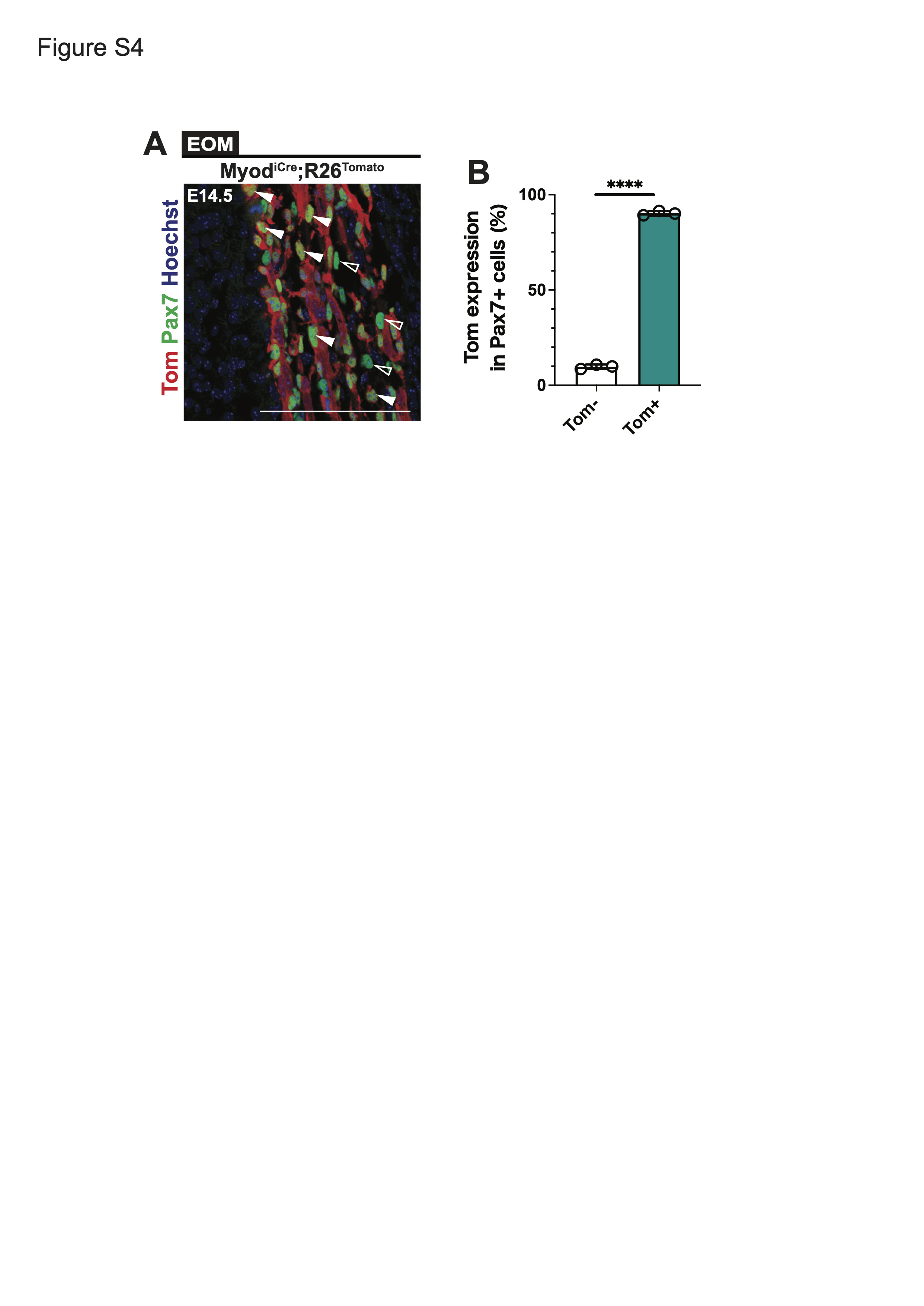

Supplement: S4 Fig — (A) Immunostaining of EOM sections from MyodiCre;R26Tomato at E14.5 for Pax7 (green), Tomato (Tom, red), with Hoechst (blue). White arrowheads indicate Tom+Pax7+ cells; open arrowheads indicate Tom-Pax7+ cells. (B) Percentage of Tom+ and negative Pax7+ cells in EOM from immunostaining in (A) (n = 3 each). Scale bar: 100μm (A). Error bars represent mean ± SEM. Two-tailed unpaired Student’s t-test. ****P<0.0001. (JPG) [file pgen.1010935.s004.jpg]

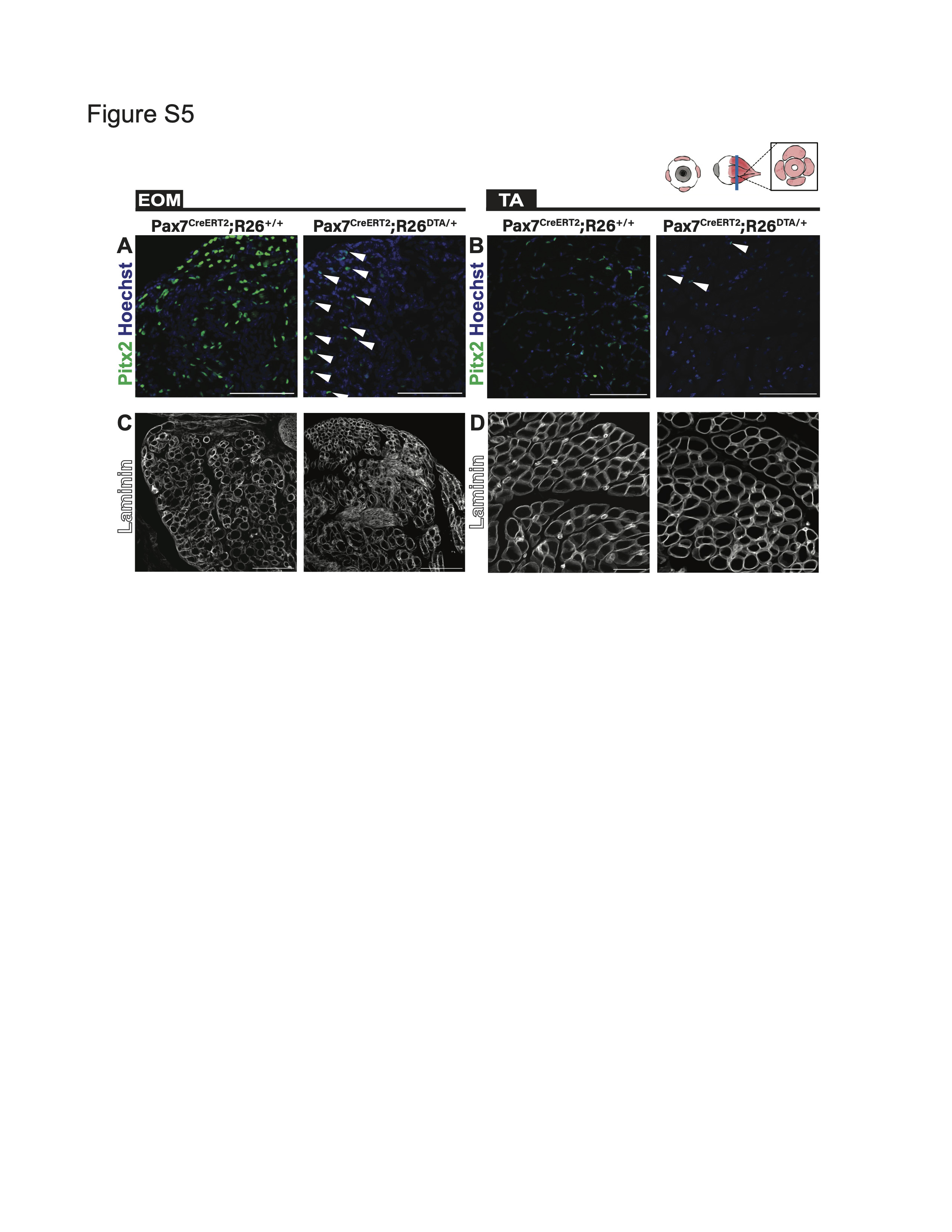

Supplement: S5 Fig — (A,B) Immunostaining for Pitx2 (green) on Pax7CreERT2;R26+/+ (control) and Pax7CreERT2;R26DTA/+ (ablated) EOM and TA sections at P8. White arrow heads indicate Pitx2+ cells. (C,D) Immunostaining for Laminin (white) on Pax7CreERT2;R26+/+ (control) and Pax7CreERT2;R26DTA/+ (ablated) EOM and TA sections at P8. Scale bars: 100μm in (A, B), 50μm in (C,D). EOM, extraocular muscle, TA, Tibialis anterior. All recti EOMs were assessed. (JPG) [file pgen.1010935.s005.jpg]

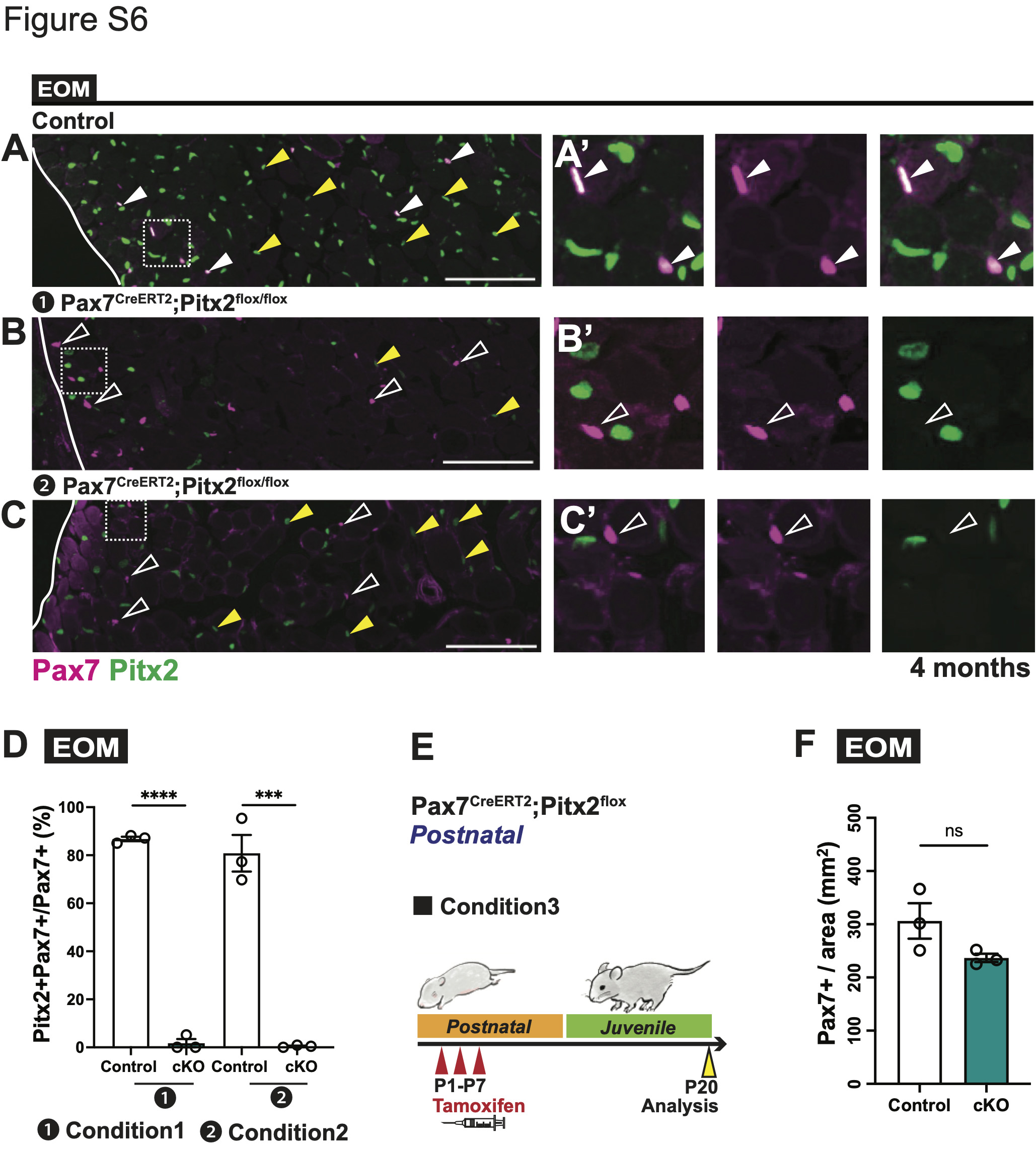

Supplement: S6 Fig — (A-C) Immunostaining of EOM sections from 4 months old Control (Pitx2flox/+) and Pax7CreERT2;Pitx2flox/flox (cKO) mice for Pax7 (magenta) and Pitx2 (green). Condition 1 (❶, induction from P1-P7, then monthly injections, samples collected at 4 months) and Condition 2 (❷, induction from P20-P25, then monthly injections, samples collected at 4 months). Higher magnification views as insets in (A’, B’, C’). White arrowheads indicate Pax7+Pitx2+ cells; open arrowheads indicate Pax7+Pitx2- cells, yellow arrowheads indicate Pitx2+ myonuclei. (D) Percentage of Pitx2+Pax7+ cells over total Pax7+ cells in EOMs from induction Condition 1 (❶) and Condition 2 (❷) (WT, cKO n = 3 each). (E) Scheme indicating timing of Tamoxifen injections for invalidation of Pitx2 with Pax7CreERT2. Condition3 (Tamoxifen induction 3 times from P1-P7, samples collected at P20). (F) Number of Pax7+ cells per area in EOM sections from Condition 3 (n = 3 each). Scale bars: 100μm (A-C). Error bars represent mean ± SEM. Two-tailed unpaired Student’s t-test. ns, non-significant, P>0.05, ***P<0.001, ****P<0.0001. EOM, extraocular muscle. All recti EOMs were assessed. (JPG) [file pgen.1010935.s006.jpg]

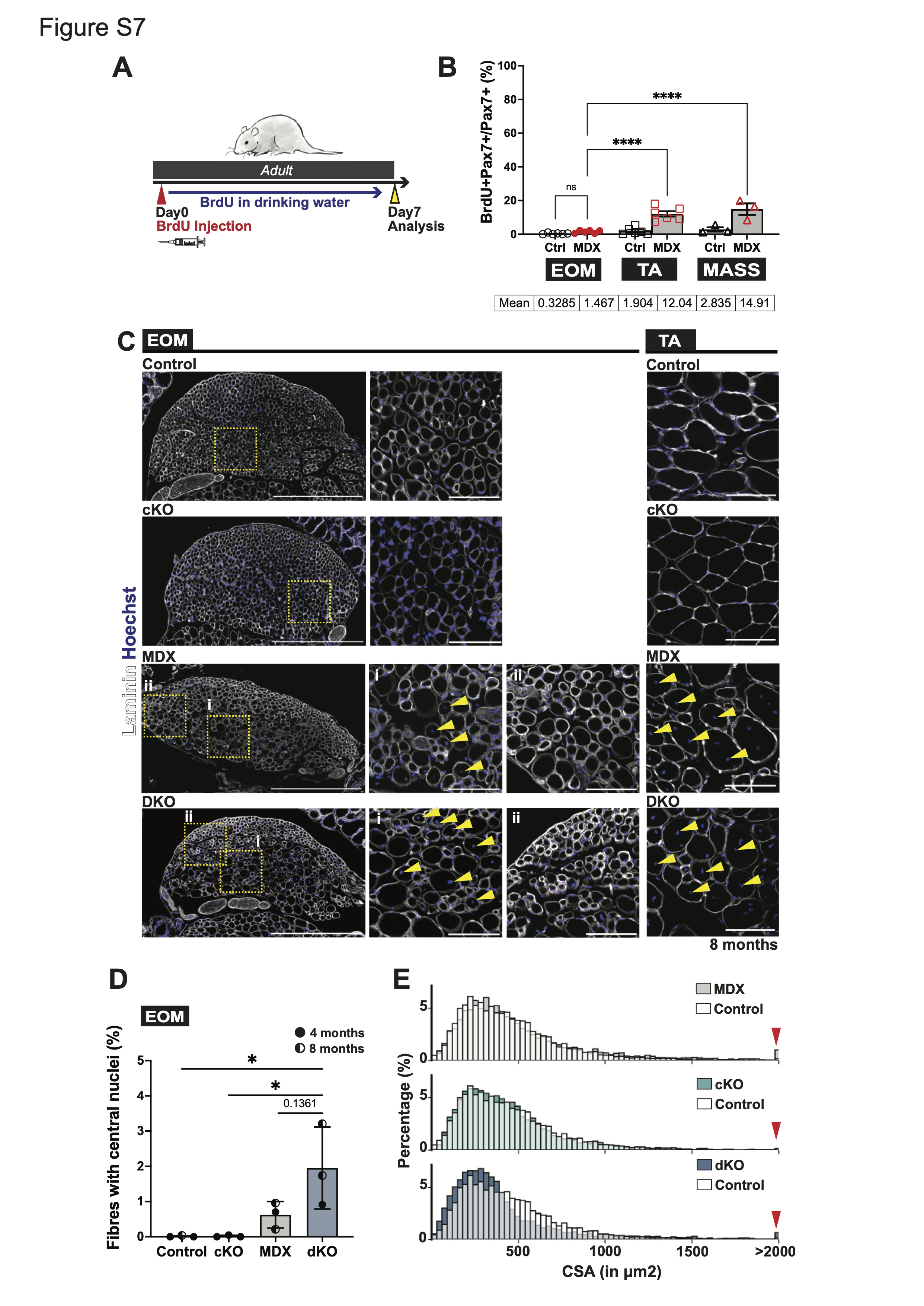

Supplement: S7 Fig — (A) Experimental scheme for BrdU uptake. A pulse of BrdU was administered intraperitoneally to adult mice followed by 7 days in drinking water. (B) Percentage of BrdU+Pax7+ cells over total Pax7+ population isolated from EOMs, TA, MASS. (C) Immunostaining of EOM and TA sections from Control (Pitx2flox/flox or Pitx2flox/+), Pax7CreERT2;Pitx2flox/flox (cKO), MDX and MDX Pitx2cKO (dKO) mice at 8 months of age for laminin (white) and Hoechst nuclei staining (blue). Right panels (i, ii), higher magnification views of the area delimited with dots. (i) Sporadic EOM regions containing central myonuclei, (ii) normal EOM regions. Yellow arrowheads indicate central myonuclei. (D) Number of centrally nucleated myofibres in EOMs from immunostaining in (C). (E) Distribution of extraocular myofibre size from immunostaining in (C) (Control, cKO and MDX, n = 3; dKO, n = 4). Red arrowheads indicate fibres with a cross-sectional area (CSA) over 2000 μm. Scale bars: (C) 1000μm in lower magnification, 100μm in higher magnification views. Error bars represent mean ± SEM. (B) Two-tailed unpaired Student’s t-test. (E) Two-way ANOVA with Dunnett post-hoc test. ns, non-significant, *P<0.05, ***P<0.001. EOM, extraocular muscle, TA, tibialis anterior, MASS, masseter. All recti EOMs were assessed. (JPG) [file pgen.1010935.s007.jpg]
