# Supplementary material for: Interplay between Pitx2 and Pax7 temporally governs specification of extraocular muscle stem cells
Source: PLoS Genet. 2024 Jun 14;20(6):e1010935. doi: 10.1371/journal.pgen.1010935 (PMC11178213; doi:10.1371/journal.pgen.1010935)

Revision for manuscript #PGENETICS-D-23-00956

New data in the revised manuscript compared to the initial manuscript
- New Figure 5 and **Supplemental Figure 5**
- Figure 5 and 6 become Figure 6 and 7 (idem for the respective supplementals)
- New panels have been added as **Main Figure 7G-K**
- New insets have been added in **Main Figure 2J,K; Figure 3D**
- New panels have been added as **Supplemental Figure S1B-D; S2N-T; S3A; S6E,F; S7D,E.**

New Data is described mainly in lines 91, 140, 191, 219, 238, 256 of the revised manuscript.
The method section has been completed.
The discussion has been extended in lines 338, 377, 410 of the revised manuscript.

Reviewer #1

General comment:

This manuscript uses a large number of genetic mutant mouse lines to understand muscle progenitor cells in the extraocular muscles compared to tibialis anterior muscles, from embryonic day E14.5 to 4 months postnatal age. I have a number of concerns that relate specifically to the way in which cells were defined, counted, and reported.

We appreciate the feedback from the Reviewer to improve the presentation of our work. We respond below to specific queries regarding the analysis and quantifications. Higher magnification insets with separate channels are now provided as part of **Figure 2J**,K. We also made available high-resolution images for the Reviewer (**Figure 1-6 for Reviewer**), one per stage analysed.

Q-1: It is always a bit worrisome when results differ from a large number of published studies from multiple laboratories. There are a huge number of genetic mice generated. There is no discussion of compensatory changes from these various manipulations. This needs to be considered. There are other mononuclear cells with myogenic potential that have been described in skeletal muscle. These also need to be considered and discussed.

- Indeed, we used a large number of genetically modified mice (one of the strengths of our laboratory) that provide complementary information for each issue addressed. These diverse approaches are concordant with each other and were needed given that the proliferation status of EOM myogenic cells is debated in the literature. **While different myogenic cell populations have been described in the literature, our study is focused specifically on the origin and proliferative status of Pax7+ MuSCs.**
- For instance, Stueltzats Dev Biol 2015 did not find Pax7+ Ki67+ EOM MuSCs right after isolation from the *in vivo* context. Pax7 is the most reliable marker of MuSCs conserved throughout the body (see Comai and Tajbakhsh, 2014; Evano and Tajbakhsh, 2018 and references therein). In many studies though, Pax7 and Ki67 or EdU co-stainings have not been performed and isolation of EOM myogenic cells were reported based on markers like CD34 (Hebert et al. Plos One 2013,

Kallestad et al. Exp Cell Research 2011). For instance, the EECD34 (CD34+/ Sca1+/CD45-/CD31-) cell population is more abundant in EOM compared to limb skeletal muscle (Kallestad et al. Exp Cell Research 2011, Hebert et al. Plos One 2013), expresses Pitx2 in vivo, but do not co-express M-Cadherin and Pax7. While these studies are informative and highlight some heterogeneity among mononuclear populations in different muscles, the question being asked in not equal given the diversity of approaches used.

To tackle the question of the different cell populations in more detail, we performed FACS analysis with surface antibodies for CD34, a7-integrin and GFP (from Tg:Pax7nGFP mice) (**Figure 7 for Reviewer**). This analysis demonstrates that the use of a single surface marker alone does not ensure specific isolation of Pax7+ MuSCs. **Thus, our study and some previous studies appear to be analysing different EOM populations, hence, these parameters require consideration to understand overall EOM biology.**

- Finally, other studies looked at EOM in species different than mouse (i.e. rabbit, McLoon et al. Muscle and Nerve 2004; monkey and human, McLoon et al. IOVS 2003) and the assumption that EOM MuSCs proliferate was based on BrdU detection on nuclei in a satellite position, but without co-staining with another marker for MuSCs such as Pax7.
- For the revised manuscript, besides Ki67 co-staining, we used EdU and BrdU pulses (**Suppl Figure S1B-D and S7D,E**) to assess proliferation of Pax7+ MuSCs in EOMs and TA.
- Regarding compensatory mechanisms, indeed this is well documented in muscle and other tissues. For example, to a certain extent Pitx2 and Pitx3 can compensate (L'Honoré Dev Biol 2007), however the Pitx2 null lacks all EOMs, and Pitx3 does not compensate in this case (Diehl IOVS 2006, Sambasivan et al. Dev Cell 2009, Zacharias et al. Dev Biol 2011). During developmental and adult myogenesis of TA muscle, the simple and double KO for these genes has been reported, again with little or partial compensation (L'honore´et al., Dev Cell 2014; L'honore´et al., elife 2018).
- The main issue of our study is whether Pax7 and Pitx2 can compensate for each other for emergence of Pax7+ MuSCs. To understand further if Pitx2 is compensated by Pax7, one strategy would be to generate Pax7; Pitx2 dKO animals (Pax7^{CreERT2}; Pitx2$^{flox/flox}$; Pax7$^{nGFP/nGFP}$). However, these experiments are heavy, involving complex breeding that would take over a year. Instead, we used a previously employed strategy of Pax7 ablation with diphtheria toxin (DTA; Murphy et al. Dev 2011, Lepper et al. Dev 2011, Sambasivan et al. Dev 2011). **New Figure 5** shows that ablation of the Pax7 population postnatally resulted in the loss of Calcitonin receptor (CalcR) + cells, and a significant loss of Myod/Myogenin double positive cells. Moreover, there was no significant increase in the interstitial Pitx2 population (Fig. S5A,B) in the ablation model, nor in *Pax7$^{nGFP/nGFP}$* null mice (Fig. S2M). This result demonstrates that an independent reservoir of myogenic cells does not compensate for the loss of the Pax7 myogenic population. We have raised this issue in the discussion.

Q-2: The Results contain a large amount of introductory material, which should be in the Introduction, and a lot of interpretation and discussion that should be in the Discussion. These should be moved to the appropriate locations.

We thank the reviewer for this suggestion to improve the reading of our manuscript. We have now moved some of this text to the introduction and discussion, however as in any manuscript style, we have short summary phrases at the end of every section to facilitate reading of the paper.

Q-3: As there are so many genetically manipulated mice, a table describing each of the mice genotypes used would help the reader.

We added a table listing the genetically modified mice as a Supplemental Table 1.

Q-4: In Figure 1E, you state that 90% of the Pax7+ cells express Pitx2. Assuming that green is Pitx2 and pink is Pax7 and co-expressing cells are white, I count 52 nuclei that are Pitx2 positive at P20, 13 nuclei that are Pax7 positive at P20, and 3 white nuclei. This would mean there were about 5% co-expressing cells, 20% were Pax7 positive, and 80% were Pitx2-positive. Similar there are even fewer Pax7 positive cells at P14 and P7. By reporting these numbers as the percent of Pax7 cells that were also Pax7+ and Pitx2+ you don't accurately represent the cell populations in your tissue samples.

Concerning the type of quantitations the reviewer proposes, we respectfully disagree with this suggestion. Performing such a quantitation based on the total number of nuclei on the image would not be informative from a myogenic angle given that it is well established in the literature that Pitx2 is expressed in the periocular mesenchyme as well (Gage et al. IOVS 2005, Comai et al. Plos Biol 2020). Our analyses address only Pitx2+Pax7+ double positive (myogenic) cells / total Pax7+ population. Here, 90% are double positive (Fig. 1D). To address the different point raised by the Reviewer, one would need a lineage tracing tool such as Pitx2^{CreERT2} that we do not have, but that can possibly be addressed in the future.

Q-5: Similarly in Figure 1C you show the percent of Pax7 positive cells that are Ki67 positive and Pax7 positive. This is not an accurate description either. If Pax7 is a marker of quiescent satellite cells, then one would expect them to be quiescent (according to Seale et al. 2000 and many other papers).

There appears to be a misunderstanding regarding Pax7 expression and quiescence/cell cycle exit. Extensive studies have shown that Pax7+ cells are in proliferation during prenatal and postnatal growth – notably, the stages examined in Fig. 1C (Fan/Zammit/Relaix/Marcelle/Tajbakhsh labs). Pax7+ quiescent cells (Ki67-negative) emerge from about P7 and it takes about 3-4 weeks for the

entire Pax7+ population to undergo cell cycle exit (Gattazzo et al. Stem Cell Reports, 2020). Therefore, Pax7+ cells are Ki67-positive or negative depending on the developmental stage examined – and this notion is coherent with the results illustrated in Fig 1C. Thus, Pax7 is a marker of satellite cell quiescence, but it also **marks historically all myogenic progenitors in development from about E13**, as well as activated progenitors after injury (reviewed in Tajbakhsh Journal of Internal Medicine 2009; Zammit & Relaix, Development 2012).

Q-6: I am very confused about the data in figure 2. If there are no Pax7 positive cells, what are the GFP-expressing cells? (although despite your counts, they sure appear to be more numerous in the sections of EOM fibers).

$Pax7^{nGFP}$ is a knock-in (KI) mouse (GFP gene inserted in Pax7 endogenous exon-sequences (Sambasivan et al., Dev Cell 2009, Dev Biol 2013). In $Pax7^{nGFP/nGFP}$ (= Pax7-null) mice, Pax7 is not expressed, but the cells are GFP+ (KI reporter gene). Similarly, in heterozygous $Pax7^{nGFP/+}$ mice, Pax7 and GFP are both expressed simultaneously. Please note also that GFP is downregulated (like the endogenous Pax7 gene) during myogenic commitment (Myod+) and differentiation (Myogenin+), where generally Pax7 and Myogenin are not co-expressed. We note that a KI/KO allele as the one we are using, inactivates the gene, allows tracking of the population but this does not necessarily imply that the cells expressing the gene will disappear, which would be the case when using DTA (diphtheria toxin ablation approach).

Q-7: On page 6, about half-way down the page (line numbers would have been helpful), you say that expression of MyoD+/Myogenin+ in cells that are GFP+ indicate “depletion of the stem cell population”. First, this is an interpretation, not a fact. I just moving these sorts of conjectures to the Discussion. Two, it is unclear why these cells are expressing these myogenic markers of differentiation. What pathway could you invoke to suggest this? Second, you don’t really mean “stem cells”, as these are not multipotent. The whole experiment described in Figure 2 is really confusing.

We have changed the text in line 117 to “depletion of the stem cell population by commitment to differentiation”. Regarding the pathway, this is the Pax7 null that is being analysed, so the absence of Pax7 itself promotes more commitment and differentiation of the myogenic population. This result agrees with previous reports following deletion of Pax7 postnatally (Lepper et al., 2009, von Maltzahn J et al. 2013; Günther et al. 2013; Relaix et al. 2006).
Regarding “stem cells” we cannot enter in detail here about the notion of stem cells, which has a vast literature, however, there appears to be a misunderstanding in this specific case. It is generally accepted that the Pax7+ (and Pax3+) myogenic cells represent “stem cells” and that the Myf5+/MyoD+ population is “progenitor/committed” in different species (ex. Rudnicki/Zammit/Tajbakhsh/Relaix/Gros/Currie/Marcelle labs, publications and reviews). Finally,

muscle stem cells (also germ-line cells) are unipotent not multipotent. Therefore, stem cells are not necessarily multipotent, and a multipotent cell is not necessarily a stem cell.

Given that GFP+ cells express Myod/Myog, this indicates that: 1) These cells are committed to differentiation, 2) The upstream Pax7+/Myod- population is depleted. As such, these are not interpretations or conjectures but data in agreement with the bulk literature in the stem cell field examining the dynamics of lineage commitment.

Q-8: Calcitonin receptor is expressed on cells involved in angiogenesis and on immune cells. It is insufficient by itself to give an identity to cells to these "formerly Pax7+ cells".

We agree that Calcitonin receptor is expressed in other cell populations. However, here we evaluate Calcitonin receptor expression in GFP+ cells – this is an important point as GFP (Pax7) is expressed only in the myogenic cells. Therefore, the identity of the cells is not in question given the specificity of this reporter (Sambasivan et al., Dev Cell 2009; used in over a dozen laboratories). Moreover, Calcitonin receptor has been widely used in the muscle field (Baghdadi et al. 2018 and references therein; and Fukada, Birchmeier, Mourikis labs).

Q-9: Similarly, you describe the population of MyoD+/Myogenin+ that are green – about ¾ of the page down - to indicate a more rapid transition from stem to committed and differentiated cells. Again your interpretation, not a fact. Move these types of statements to the Discussion.

We respectfully disagree with the Reviewer regarding this observation. Given that these myogenic commitment and differentiation markers are more elevated in the null compared to the heterozygous (both being GFP+, see above), the most parsimonious explanation is that the stem cell population in the null (Pax7+ and/or GFP+) has a greater propensity to differentiate with a progressive reduction in the number of upstream cells. The interpretation of such a phenomenon is not unusual as similar observations and conclusions have been made in other contexts (ex. Lepper et al. 2011). This reasoning was used also by the Rudkicki/Fan/Relaix/Braun labs when examining the Pax7 null in limb muscles. In all cases the stem/progenitors exhibited accelerated differentiation, therefore our results are coherent with those from different labs.

Q-10: In Figure 2J, K, and L, again you are assessing the percent of green cells that are green and pink. From the images, however, it is clear that as a percent of pink cells, there aren't very many green ones. Please represent the data to more accurately depict the actual data. There are actually very few pitx2 positive cells that also express the GFP, and in TA there are almost no green cells whatsoever.

We thank the Reviewer for pointing this out. Please note similar reasoning for countings in Fig 1E. Pitx2 intensity in TA is much lower than in the EOM, but detectable broadly. We have now added separate channels of the insets for these panels to highlight this point in Fig. 2J,2K.

Q-9: In figure 3, you indicate that "virtually all" of the cells express both Myf5 and Pitx2. Please show an overlay of the Pitx2 and beta-gal staining at sufficient power where cells can be seen.

A-9:
We have included a higher resolution image with the separated channels in this Figure (Fig. 3D) and rephrased the text as "most Myf5+ cells express Pitx2" (Line 159).

Q-11: In Figure 3D and E, I don't see that all the Pax7+ cells were also beta-gal positive. I see mostly green cells, some pink cells, but I don't see them co-expressing (as I assume they would then be white.) Is this image not typical?

As with the previous points regarding co-expression, if the intensities are not similar, the overlapping color is not homogeneous. To illustrate this point, we provide higher magnification images as **Supplemental Figure S3A** as well as a composite for all stages for the reviewer (**Figure 8 for reviewer**).

Q-12: Page 9, paragraph 3 line 6: change "depletion" to "decrease" since there appear to be pitx2 positive cells still present.

It is not clear to which paragraph the reviewer is referring to. No "depletion" is written in Page 9 Paragraph 3 line 6 of the first version. It appears in Page 6. If the reviewer is actually referring to the word "deletion" indeed present in Page 9 Paragraph 3 line 6, it is worth considering the following: It is the deletion of the Pitx2 gene which is efficient, i.e. no more Pitx2 expression in the Pax7+ lineage due to the genetic deletion with CreERT2. The remaining green cells are interstitial and Pax7-negative or myonuclei.

Q-13: In the Discussion, page 12, paragraph 2, please add in the appropriate other references that loss of Pitx2 results in loss of extraocular muscles (Zacharias et al., Diehl et al.) and add the references further down that same page that show that MyoD is needed for progression along the myogenic differentiation lineage (e.g. Zacharias et al 2011; L'Honore et al. 2010), etc.

We thank the reviewer for these comments. Concerning the references, all references related to EOM loss in Pitx2 mutants have been cited in the introduction (line 37).
In the discussion we refer to:

“*Pitx2* is critically required cell-autonomously to maintain myogenic progenitors in an immature state, ensure their survival, and to activate the MRFs. This is exclusively addressed by (Zacharias et al., 2011)” → Diehl 2006 analyzed a full mutant, so including this reference here would be misleading. “*Pitx2* expression persists following activation of the MRFs and myogenic commitment in the developing EOMs, and it is required for myogenic cell survival until the end of the embryonic period (Sambasivan et al., 2009).” → L’Honoré 2010 did not study this regulation in the EOMs.

Q-14: The Methods state that significance was determined by Students’ t-tests. You cannot use Students’ t-tests on data with more than 2 comparisons e.g. Figure 1C, 1D, Figure 2 C, F, I, L, Figure 3E, Figure 5C, D, F, G, Figure6, C, D, E, F, and in the bar graphs of the supplemental figures. These need to be redone using ANOVA and posthoc t-tests if significance is shown.

We thank the reviewer for raising this point. Indeed, the statistical test to use depends on the biological question that we ask. When we are interested in comparing only two conditions (within a same muscle), the appropriate test is a t-test. When looking at differences between multiple groups and identifying which groups actually differ, an ANOVA with post-hoc tests should be used. We have performed ANOVA and posthoc t-tests except when straight pairwise comparisons were performed. Figure legends have been changed accordingly.

Reviewer #2

General comment:

This is a well written manuscript with great presented research. Most of my comments are minor and many of them are stylistic.

We thank the Reviewer for recognising the importance of our work and their suggestions.

Q-1: Consider adding to the introduction a few sentences about the EOMs. It is mentioned in Fig 1 that there are 4 recti and 2 obliquus but it would be helpful to mention this also in the introduction.

We thank the Reviewer for this suggestion. We added this to the introduction.

Q-2: I was wondering if there were any differences in the results between the EOMs as they derive from multiple myogenic sources. I haven't seen any differences in the pictures. Perhaps a sentence or two could be added in the results to address this shortly, even if it is just 'There were no differences".

This is an important point raised by the Reviewer. We have not seen differences between individual quantifications performed in each rectus muscle, and at their medio-lateral attachment sites (base of the skull and orbit). Unfortunately, a mouse model to track prechordal versus cranial paraxial

mesoderm is not yet available to fully answer this question. We added a phrase in the legend saying that all recti EOMs were analysed, in the methods section and in line 107.

Q-3: A few abbreviations were not introduced before 1st time use.

Corrected

Q-4: Some abbreviations should be updated in context of the text. These are suggestions:
4A. EOM MuSCs change to EO MuSCs (introduce EO) as M and Mu are both muscle
4B. EOM myofibers change to EO myofibers as muscle myofibers is redundant in my opinion
4C. EOM and TA muscles change to EOMs and TA or to EO and TA muscles

These are interesting suggestions by the Review that we have considered. Talking about Tibialis anterior leaves implicit the notion of “muscle” while extraocular muscle has “muscle” as part of the name. We are somewhat obliged to retain the nomenclature to be consistent with our past papers, nevertheless we rephrased the sentences highlighted by the Reviewer to avoid the redundancy noted. For example, we changed EOM MuSCs to MuSCs in the EOM or extraocular MuSCs in most cases.

Q-5: Use either fetus or foetus but not both in the manuscript.

Corrected.

Q-6: Consider adding abbreviation explanations to the Figure legends so that they can stand alone (independent of the manuscript).

Abbreviations were added at the end of each figure legend.

Q-7: Consider changing EOM to EOMs in all figures as most of them show more than 1 EOM.

As indicated above, we retained some of our nomenclature to be coherent with our past papers in the figure but adjusted the legends to be clear that we are analysing all the recti muscles. Yablonka-Reuveni, McLoon, our lab have been using EOM, while Relaix lab has recently moved to EOMs.

Q-8: Throughout the text are several occasions where EOMs would fit better than EOM. I added the s where I thought it would be appropriate but leave it to the authors to decide if they want to keep it or not.

We thank the Reviewer for having taken the time to make these suggestions, most of which have been incorporated.

Q-9: Other very minor things were changed using track changes in the attached PDF

We thank the Reviewer for carefully reading the manuscript and for their suggestions.

Reviewer #3

General comment:

This manuscript by Kuriki et al. described the stage specific role of Pitx2 and Pax7 in the specification of EOM progenitors during muscle development and postnatal muscle growth, based on a series of immunohistological assays. Although EOM is well known as a sparing muscle for pathology of muscular dystrophy, the underlying mechanism is largely unknown. Using Cre-driver mouse lines, the authors show that Pitx2 is required to generate (or maintain) myogenic progenitors in the developmental stage but not in the postnatal and juvenile stages. The number of muscle stem cells in EOM is more significantly reduced by Pax7 inactivation compared to those of TA muscle. EOM progenitors tend to enter a quiescent state earlier than those in TA muscles in the postnatal stages. Pax7+ cells are less proliferative in EOM of mdx mice compared to those of TA muscle, which is probably in a Pitx2-independent manner. Overall, this study further characterizes the heterogeneity of muscle stem cells among muscles, providing new insights into pathogenesis of muscular dystrophy. Although the findings are very interesting and the manuscript is well-written, there are some points that need to be mentioned or discussed before publication.

We thank the Reviewer for recognising the importance of our work and their suggestions.

Q-1: Figure2: GFP-expressing cells in EOM, but not TA, tend to commit to myogenic differentiation when Pax7 is removed. Is increased apoptosis involved in Pax7-KO EOM cells at P20?

Indeed, this is an important point. To address this issue in $Pax7^{nGFP/nGFP}$ null cells, we performed immunofluorescence staining for cleaved Caspase-3 at E14.5, P1 and P20 (Supplemental Figure 2N-T). We observed an increased number of Cleaved caspase 3 at P1. This result is now discussed in the context of previous data on Pax7 null in the trunk (Relaix et al., 2006).

Q-2: Figure 2K: an arrowhead is mislocated.

Corrected.

Q-3: Figure 5: in Figure 5A-D, did the authors check whether the decrease in the Pax7+ cell numbers persists in the postnatal muscle growth and adult stages (e.g., in 2-month-old mice)? In Figure 5E-G, how about Pax7+ cell numbers at the earlier time points after TAM treatment (e.g., at day 5 after TAM)? Please discuss these points.

- For Figure 5A-D (new Figure 6A-D): We made great efforts to generate Pax7^{CreERT2}; Pitx2^{flox} KO mice induced at E11.5+E12.5 to be analyzed postnatally. However, we did not obtain living pups even when progesterone was co-administered. This is a common problem in the field as tamoxifen injected during the embryonic period can cause abortions and mortality of pups at birth due to difficult delivery (Ved et al. Lab Anim 2019, Lizen et al. Transg Research 2015).
- Concerning Pax7 numbers at earlier time points after TAM treatment, we have now quantified Pax7+ cells 2 weeks after induction of Pitx2 recombination (Suppl Figure S6E-F). No change in the number of Pax7+ cells/area was observed indicating that this is the "the facto" status and no compensation occurred when samples were analyzed at later time points post tamoxifen induction (Figure 6E-G).

Q-4: Figure 6: the Pax7+cell population of the mdx mice was evaluated with mixed ages ranging from 4 to 12 months of age. I guess that the authors used as many mdx mice as possible for this study. Are there any aging effects on EOM Pax7+cells of mdx mice? If so the authors should carefully interpret these results. Please discuss this point.

We agree with the reviewer that mixing stages is not a good strategy, especially for mdx phenotype. In the first version we had included 4 months old mice to have enough N values. For the revisions, we obtained more samples at 8 months and 1year of age and plotted them individually.
Concerning the ageing effect of EOM Pax7+ cells in mdx vs control mice, we have now added new data as Figure 7G-J. In both EOM and TA, mdx and control, there was a progressive reduction in the number of Pax7+ cells/area with age. Yet, in the TA of mdx mice, the number of Pax7+ cells/area showed the opposite trend in muscle areas showing damage. Thus, even at late stages of the disease, the MuSCs in the EOM appear not to proliferate in the mouse.

Q-5: Figure 6: EOM is well known to be spared in Duchenne muscular dystrophy. The authors found that muscle stem cells are less proliferative in EOM of mdx compared to those of limb muscle. Is this phenomenon related to a recent study revealing that depletion of muscle stem cells attenuates pathology in muscular dystrophy (Nat Commun 2022 May 26;13(1):2940)?

We thank the Reviewer for raising this point. The authors of that study proposed that reactivation of the foetal program via Myod might be provoking destabilisation of myofibres in mdx mice. Following this logic, one possibility is that less proliferation in EOM limits destabilisation of mdx myofibres by minimising the input of Myod+ myoblasts into the myofibre. We added this study and addressed this point in the discussion line 410.

Q-6: Mouse strains in Methods: "mdx-βgeo (Wertz and Füchtbauer, 1998)" is duplicated.

Corrected.

Q-7: Option: a schematic summary of the findings might be helpful for readers' understanding.

We appreciate this suggestion. We put a scheme as a Figure 7K.

Figures for Reviewers

FIGURE 1. Immunostaining for Pax7 (red) and Pitx2 (green) on embryonic day (E) 14.5 cryosections at the EOM level. Nuclei stained with Hoechst (blue). Higher magnification views of the area delimited with white line. White arrowheads indicate Pax7/Pitx2 double positive cells.

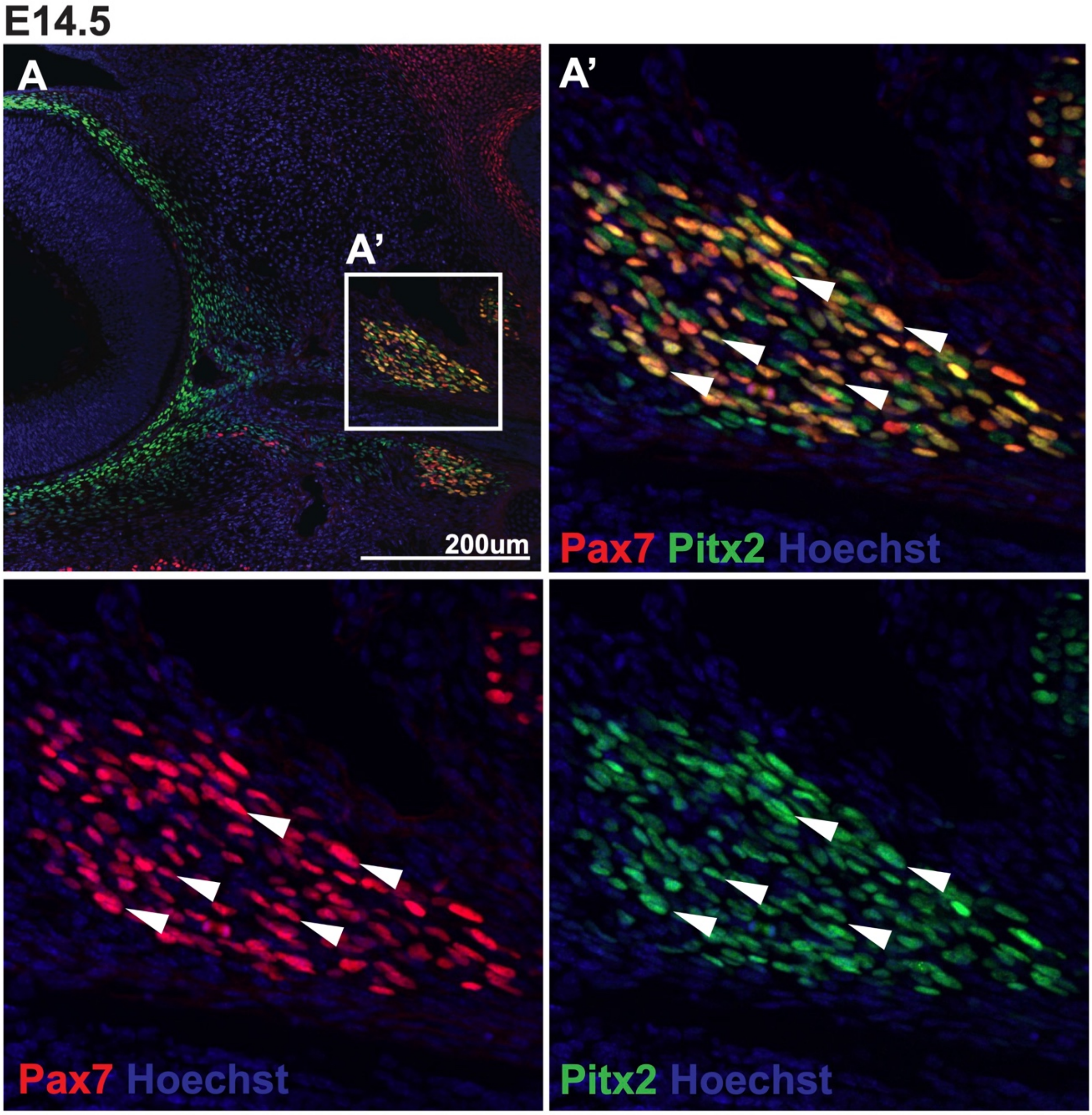

FIGURE 2. Immunostaining for Pax7 (red) and Pitx2 (green) on embryonic day (E)17.5 cryosections at the EOM level. Nuclei stained with Hoechst (blue).

Higher magnification views of the area delimited with white line. White arrowheads indicate Pax7/Pitx2 double positive cells.

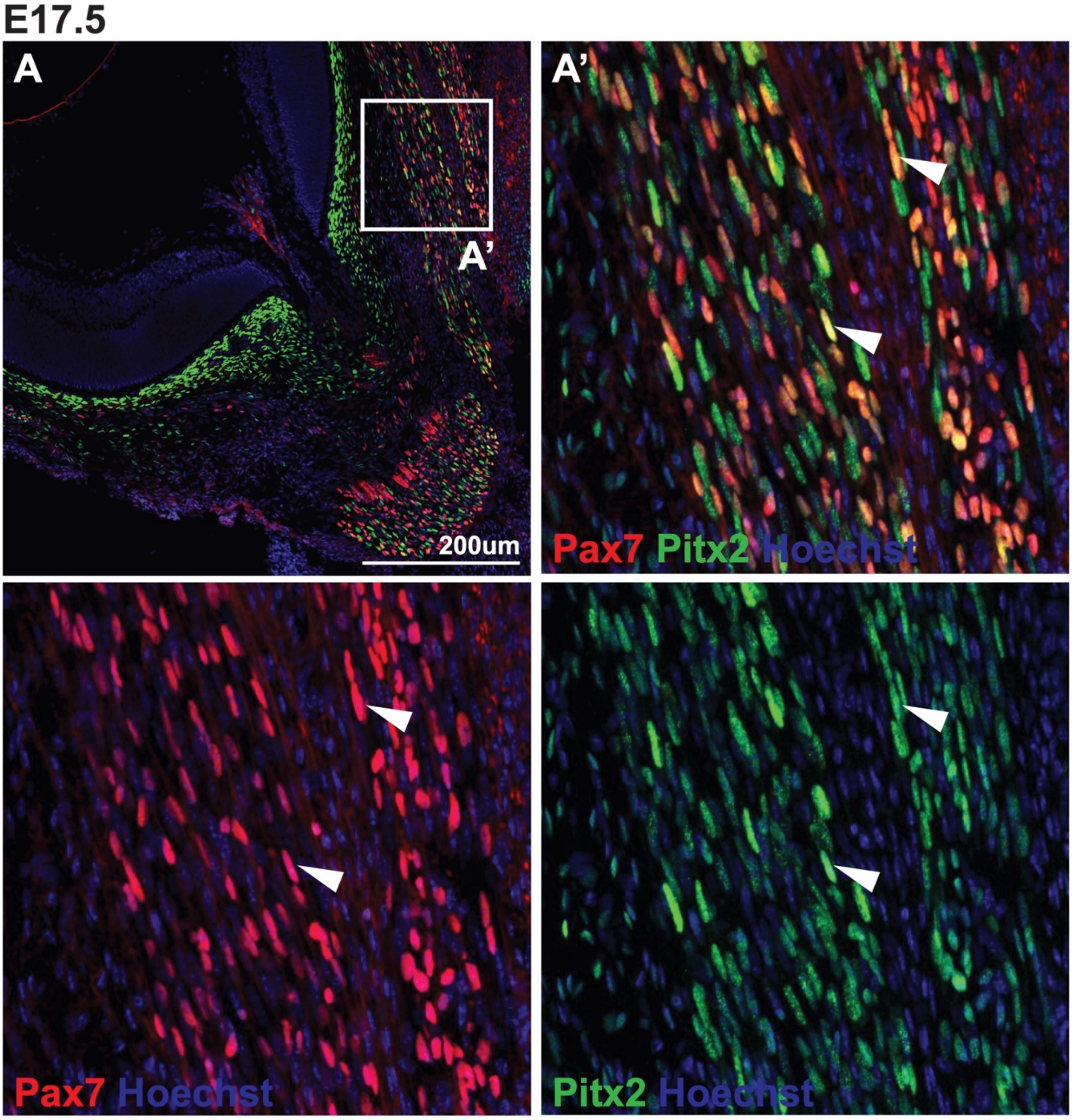

FIGURE 3. Immunostaining for Pax7 (red) and Pitx2 (green) on postnatal day (P) 7 EOM cryosections. Nuclei stained with Hoechst (blue). Higher magnification views of the area delimited with white line. White arrowheads indicate Pax7/Pitx2 double positive cells.

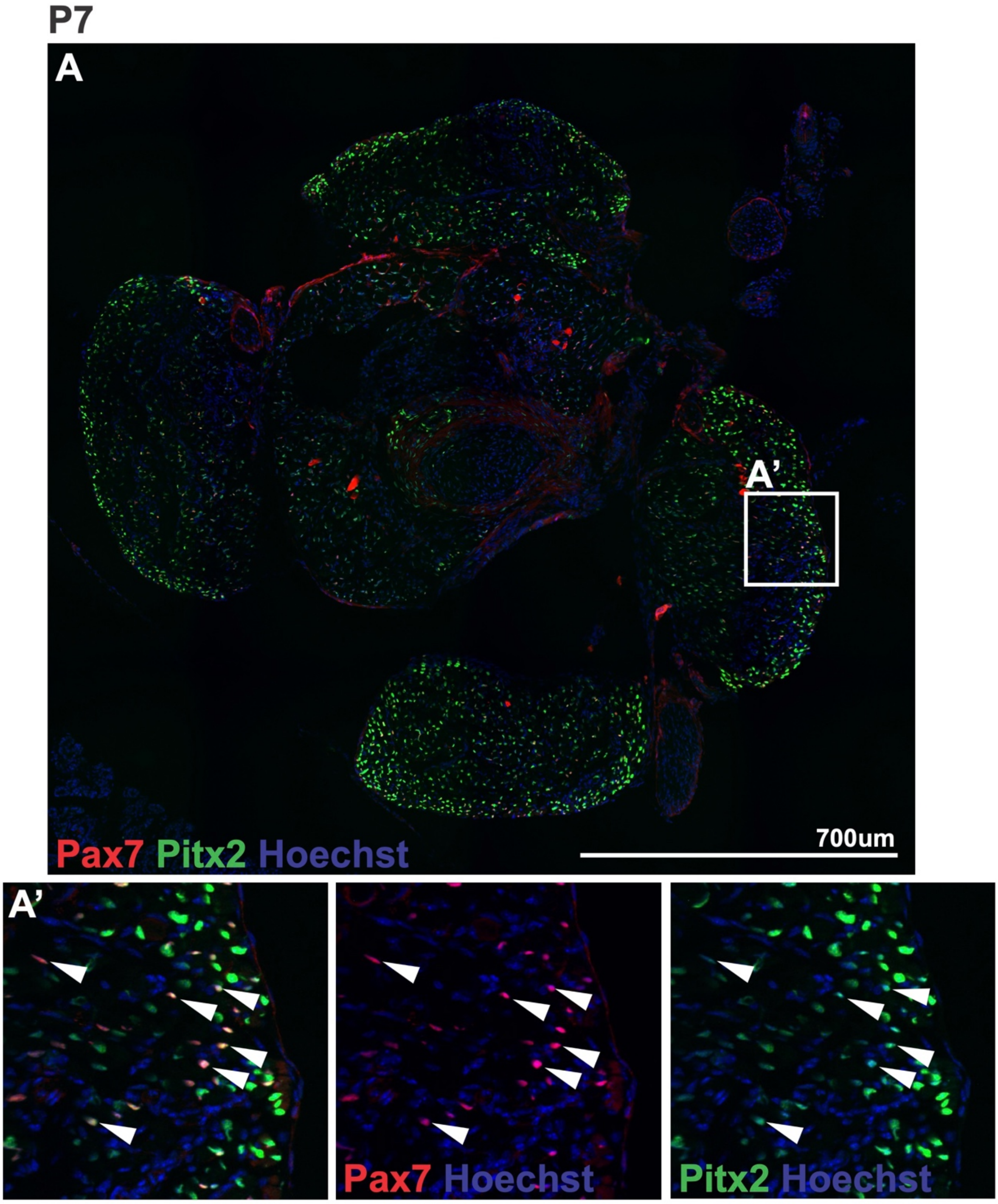

FIGURE 4. Immunostaining for Pax7 (red) and Pitx2 (green) on postnatal day (P) 14 EOM cryosections. Nuclei stained with Hoechst (blue). Higher magnification views of the area delimited with white line. White arrowheads indicate Pax7/Pitx2 double positive cells.

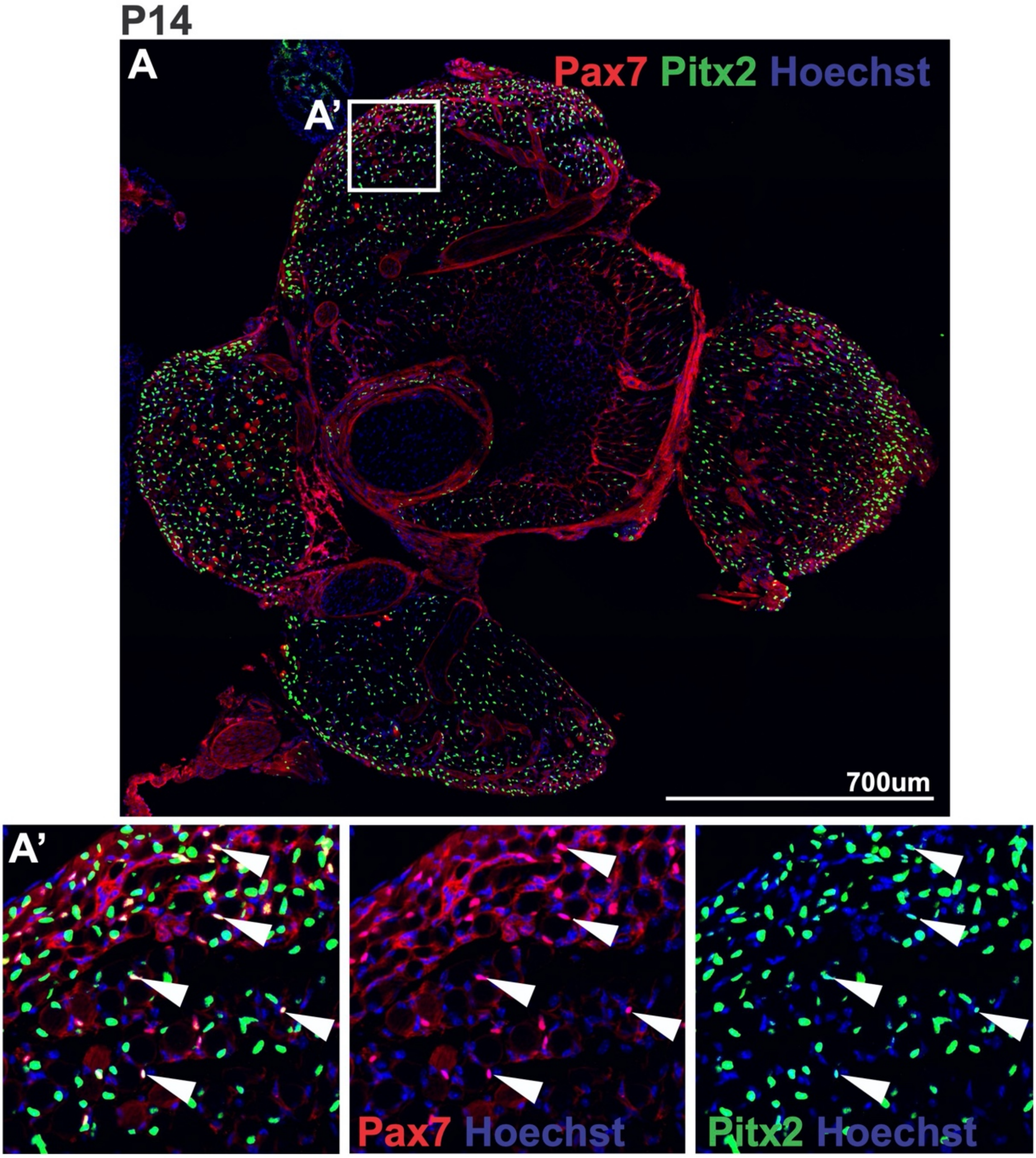

FIGURE 5. Immunostaining for Pax7 (red) and Pitx2 (green) on postnatal day (P) 20 EOM cryosections. Nuclei stained with Hoechst (blue). Higher magnification views of the area delimited with white line. White arrowheads indicate Pax7/Pitx2 double positive cells. On note, there was no Pitx2 expressing cells in the Harderian gland (A''), showing specific Pitx2 expression pattern in EOMs.

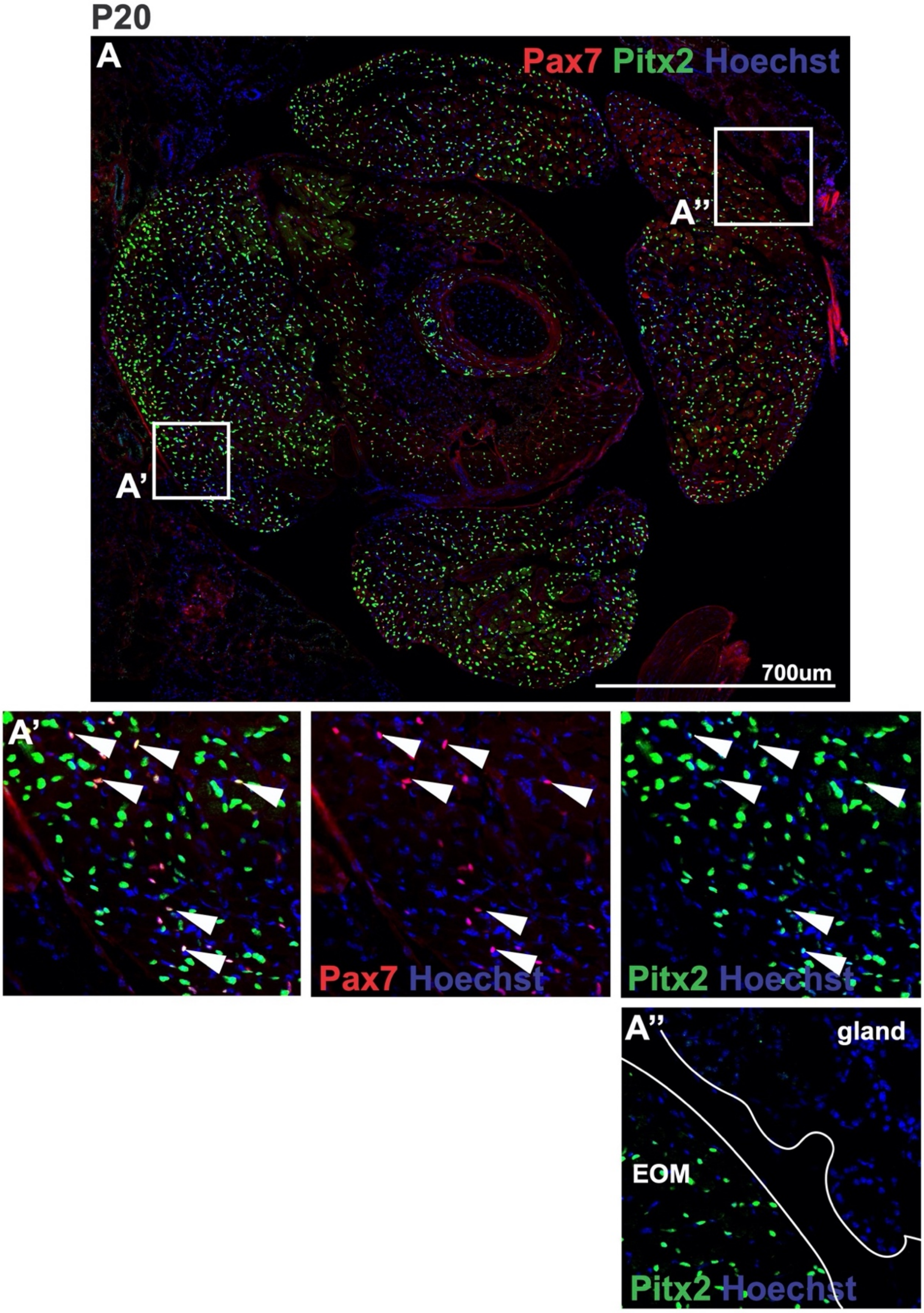

FIGURE 6. Immunostaining for Pax7 (red) and Pitx2 (green) on adult (4 months old) EOM cryosections. Nuclei stained with Hoechst (blue). Higher magnification views of the area delimited with white line. White arrowheads indicate Pax7/Pitx2 double positive cells.

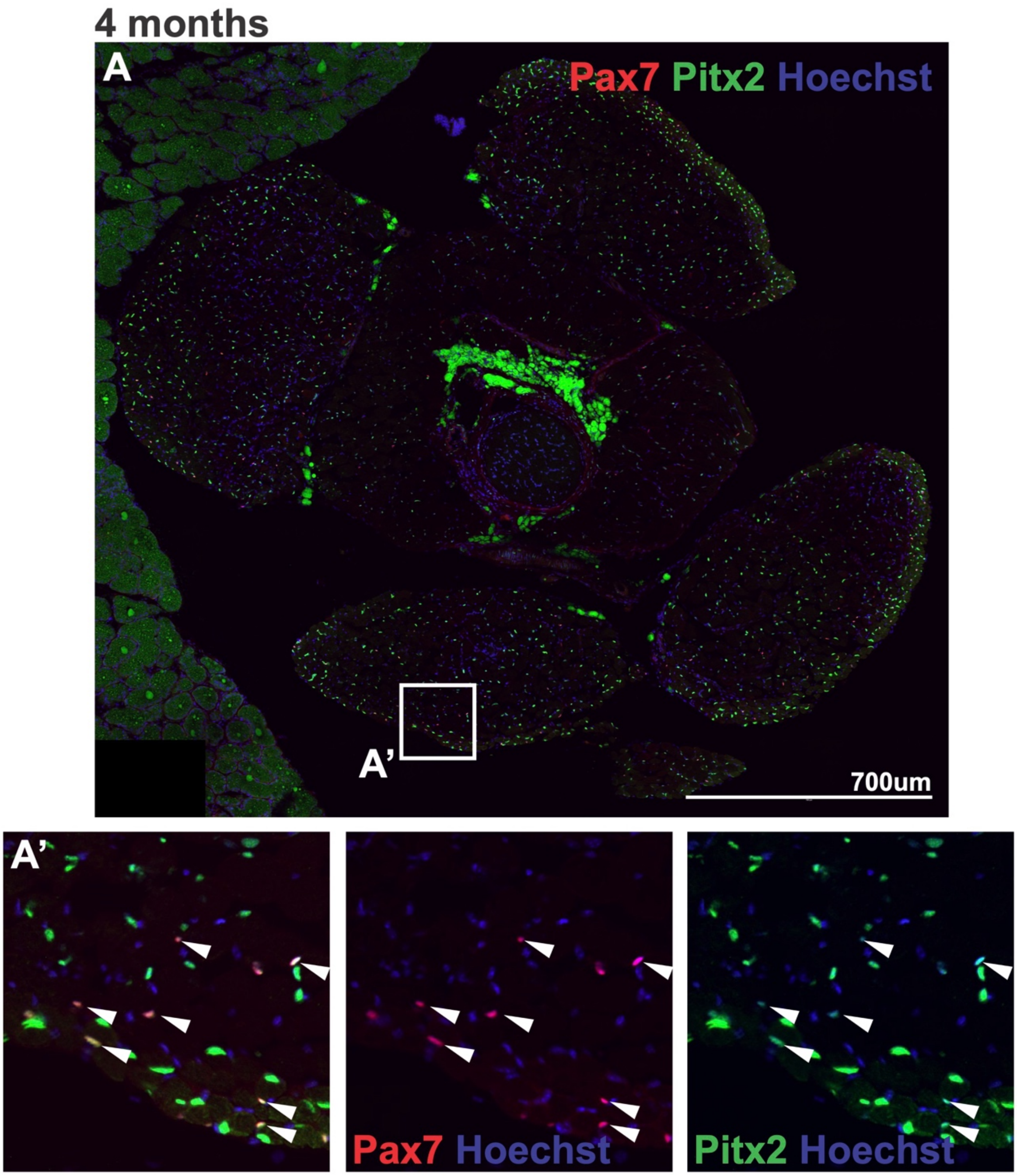

FIGURE 7. FACS analysis of EOM subpopulations. **A.** Gating strategy. CD31-CD45-SCA1- cells were analyzed in terms of CD34, a7-integrin and GFP. **B-C.** EOM and TA profiles. Note that not all CD34 or a7-integrin cells are GFP+ (blue circles). As formerly shown (Kallestad 2011, Hebert 2013), the EOM has a larger fraction of CD34+ cells than the TA (red circles).

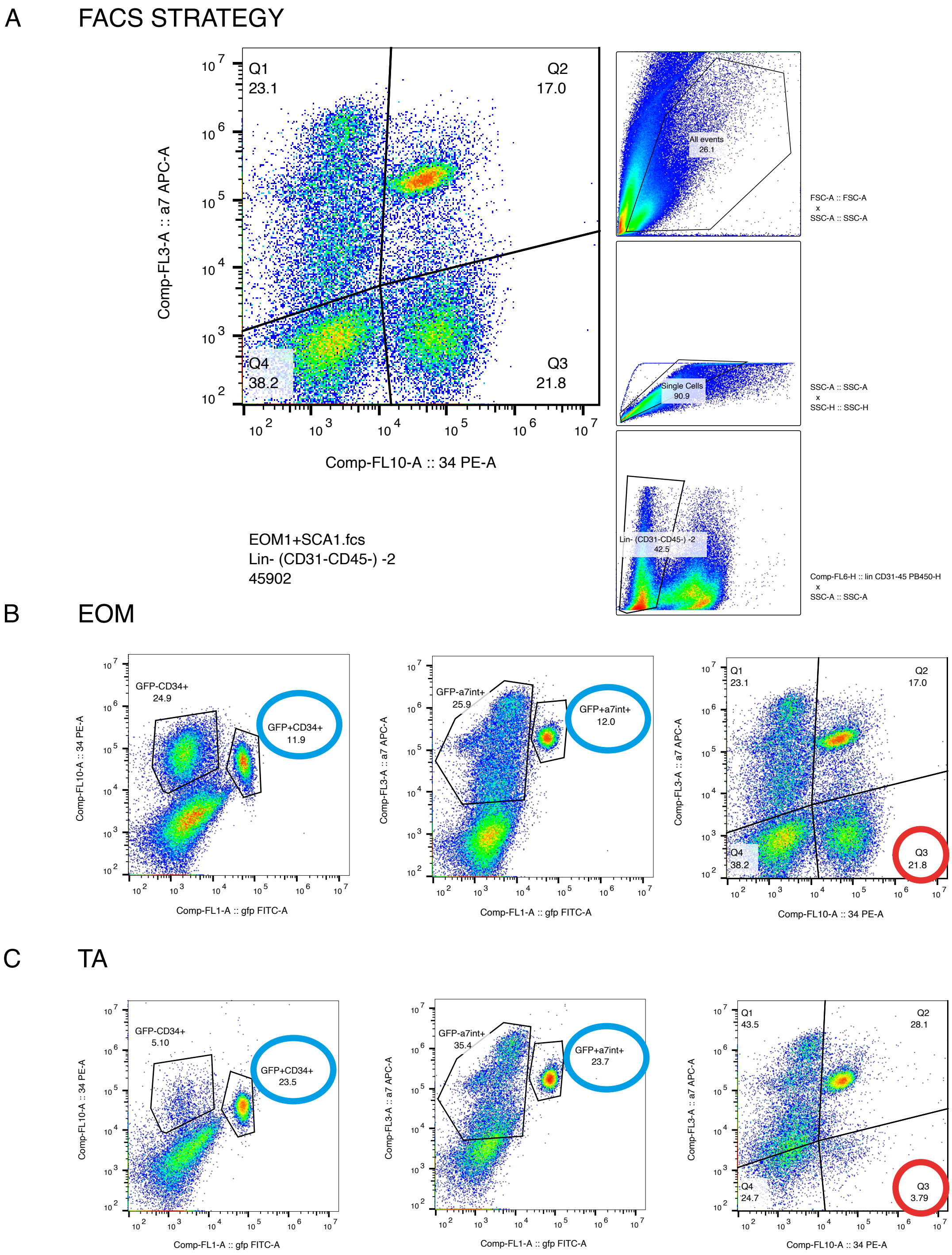

FIGURE 8. Single Z-plane of whole mount immunostaining for βgal (*Myf5*^{nlacZ}) (green) and Pitx2 (magenta) at embryonic day (E) 10.5, E11.5, E11.75 and E12.0 highlighting co-localisation of both markers at every stage analysed.

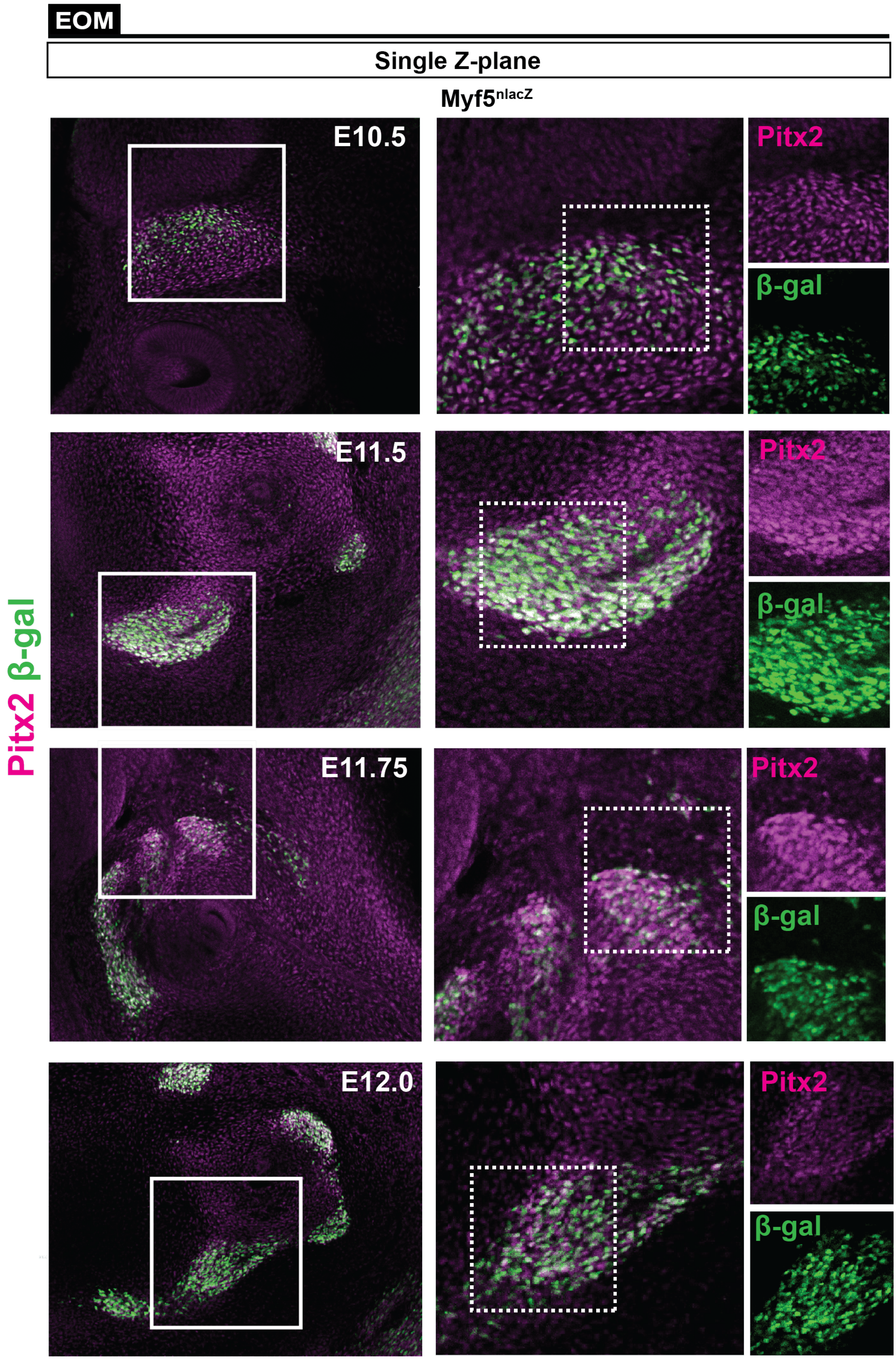

Supplement: Attachment — Submitted filename: Text_Figs_reviewer_V12.pdf [file pgen.1010935.s011.pdf]
